# A *Streptococcus* Quorum Sensing System Enables Suppression of Innate Immunity

**DOI:** 10.1128/mBio.03400-20

**Published:** 2021-05-04

**Authors:** Kate M. Rahbari, Jennifer C. Chang, Michael J. Federle

**Affiliations:** a Department of Microbiology and Immunology, University of Illinois at Chicago, Chicago, Illinois, USA; b Department of Pharmaceutical Sciences, University of Illinois at Chicago, Chicago, Illinois, USA; Carnegie Mellon University

**Keywords:** immunosuppression, innate immunity, macrophage, pheromone, biosynthetic gene cluster, NF-κB, TLR, cytokines, macrophages

## Abstract

Some bacterial pathogens utilize cell-cell communication systems, such as quorum sensing (QS), to coordinate genetic programs during host colonization and infection. The human-restricted pathosymbiont Streptococcus pyogenes (group A streptococcus [GAS]) uses the Rgg2/Rgg3 QS system to modify the bacterial surface, enabling biofilm formation and lysozyme resistance. Here, we demonstrate that innate immune cell responses to GAS are substantially altered by the QS status of the bacteria. We found that macrophage activation, stimulated by multiple agonists and assessed by cytokine production and NF-κB activity, was substantially suppressed upon interaction with QS-active GAS but not QS-inactive bacteria. Neither macrophage viability nor bacterial adherence, internalization, or survival were altered by the QS activation status, yet tumor necrosis factor alpha (TNF-α), interleukin 6 (IL-6), and interferon beta (IFN-β) levels and NF-κB reporter activity were drastically lower following infection with QS-active GAS. Suppression required contact between viable bacteria and macrophages. A QS-regulated biosynthetic gene cluster (BGC) in the GAS genome, encoding several putative enzymes, was also required for macrophage modulation. Our findings suggest a model wherein upon contact with macrophages, QS-active GAS produce a BGC-derived factor capable of suppressing inflammatory responses. The suppressive capability of QS-active GAS is abolished after treatment with a specific QS inhibitor. These observations suggest that interfering with the ability of bacteria to collaborate via QS can serve as a strategy to counteract microbial efforts to manipulate host defenses.

## INTRODUCTION

Bacteria possess elegant mechanisms to subvert host recognition and overcome the multiple barriers of human immune defenses. Strategies for survival have evolved to incorporate complex signaling systems that regulate genetic programs necessary to adapt to multiple environments and threats encountered in the host. Cell-cell communication systems, such as quorum sensing (QS), allow bacteria to disseminate information about their environment, density, and metabolism through the transmission of extracellular signals. QS systems invoke social pressures that result in the synchronization of genetic programs across the population. This synchronization enables bacteria to function as a multicellular group better equipped to perform activities such as forming biofilms, producing virulence factors, or taking up foreign DNA (1).

The bacterium Streptococcus pyogenes (group A streptococcus [GAS]) is a serious pathogen responsible for a high global burden of disease, including 616 million annual cases of GAS pharyngitis and 1.78 million annual cases of severe diseases like necrotizing fasciitis, toxic shock syndrome, rheumatic heart disease, and glomerulonephritis (2). GAS utilizes several QS systems, including RopB (Rgg1), Rgg2 or Rgg3 (Rgg2/3), ComR (Rgg4), and Sil. These systems have been shown to regulate virulence factors, biofilm production, competence genes, and invasive disease, respectively (1). Notably, the Rgg2/3 system is conserved across all serotypes of GAS, indicating its importance for GAS survival and making it an attractive system to study.

The transcriptional regulators Rgg2 and Rgg3 respond to two functionally equivalent short hydrophobic peptide ligands (SHP2 and SHP3, collectively referred to as SHP) in a concentration-dependent manner (3, 4). When concentrations of SHP are low, the repressive activity of Rgg3 predominates at target promoters. When SHP reaches a critical concentration (∼1 nM in culture conditions), it binds to Rgg2 and Rgg3, causing inactivation of Rgg3 (leading to derepression of target genes) and activation of Rgg2 (leading to transcriptional activation of target genes). Genetic tools have helped identify two genetic loci that are directly controlled by the Rgg2/3 system: (i) the region adjacent to and including *shp2*, including *spy49_0414c* (*stcA*) and (ii) the region adjacent to and including *shp3* and *spy49_0450-0460*. Rgg2/3-mediated regulation of *stcA* promotes aggregation and biofilm development (two phenotypes that require adhesive surface structures) and resistance to lysozyme (an antimicrobial host factor that targets components of the bacterial cell wall) (3, 5). Because the surface of GAS interfaces with host cells, we hypothesized that Rgg2/3-mediated alterations to GAS would result in altered host immune responses.

Early innate immune responses to GAS are critical for control of infection (6). Pharmacological and genetic depletion studies established the importance of macrophages, dendritic cells (DCs), and neutrophils in initiating the innate immune response against GAS via distinct pathways (6–9). Macrophages are particularly important, as *in vivo* depletion or blockage of phagocytosis drastically enhance susceptibility of mice to GAS (6, 7). In response to GAS, macrophages upregulate expression of genes involved in inflammation, survival, and production of oxygen radicals, which help them eliminate GAS efficiently (10). Signaling through MyD88 in macrophages and dendritic cells is required for cytokine responses and GAS clearance, illustrating the importance of pathogen sensing through Toll-like receptors (TLRs) and interleukin 1 receptor in response to infection (11–14). Downstream of MyD88, a cascade of events leads to the translocation of NF-κB family proteins to the nucleus, where they regulate expression of genes required to promote inflammation, including cytokines, adhesion molecules, and cell growth or death factors.

Because the Rgg2/3 QS system alters surface properties of GAS, this study aimed to determine the consequence on immune responses using an *in vitro* infection model. Macrophages were infected with isogenic GAS mutants lacking either the transcriptional repressor, *rgg3* (QS-locked-ON) or the transcriptional activator, *rgg2* (QS-locked-OFF). Here, we report that Rgg2/3 activation limits proinflammatory cytokine responses to GAS. Moreover, QS activation resulted in suppression of cytokine responses induced by several TLR agonists. Expression of the putative biosynthetic gene cluster (BGC) downstream of *shp3* was found to be required for this phenotype. Overall, this study has identified a new role for Rgg2/3 QS in suppressing cytokine responses to GAS *in vitro*.

## RESULTS

### Macrophage responses to GAS are attenuated when Rgg2/3 QS is active.

**FIG 1:**
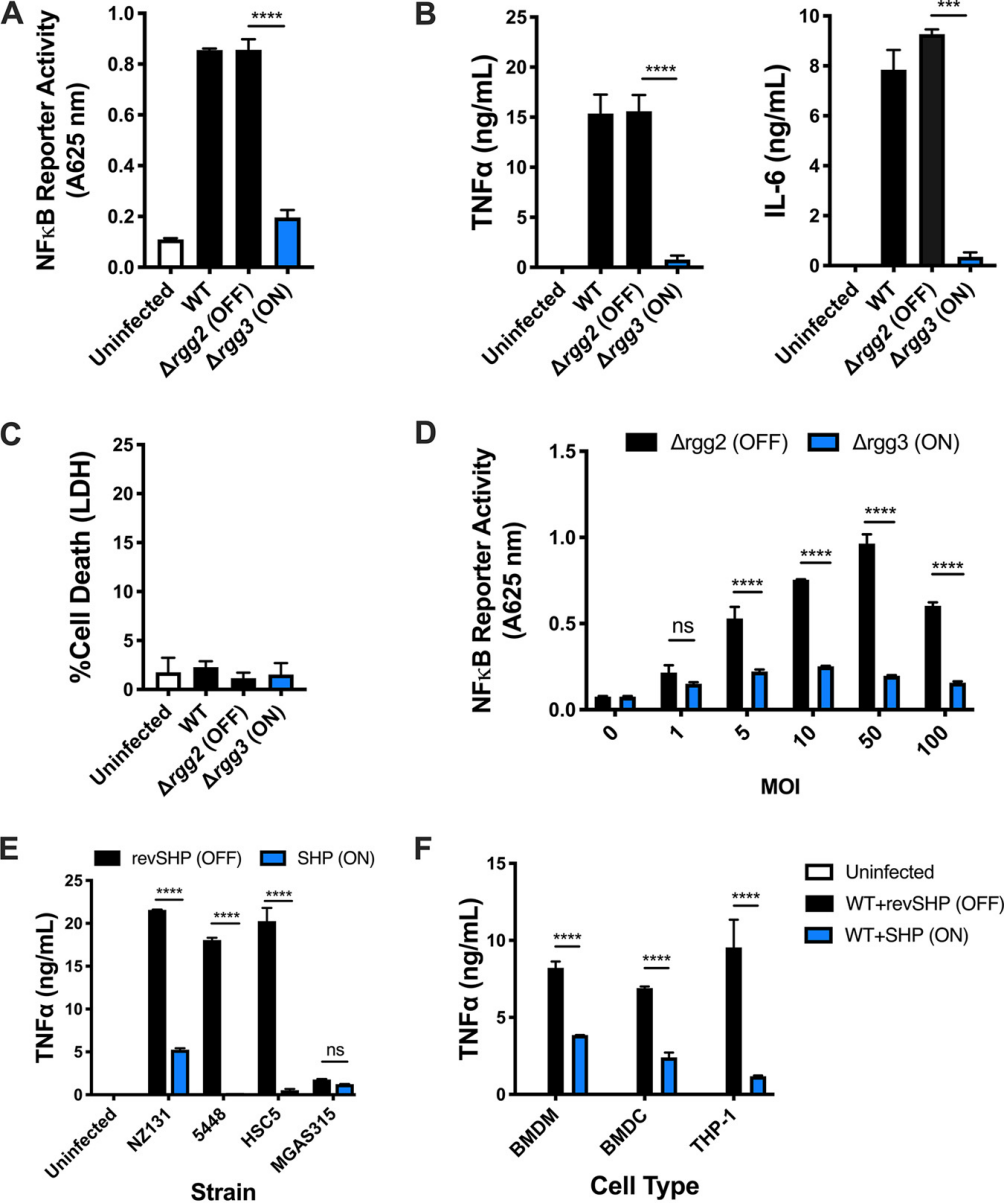
Macrophage responses to GAS are attenuated when Rgg2/3 QS is active. (A) NF-κB responses after infecting RAW246.7 cells containing a chromosomally integrated NF-κB-inducible secreted embryonic alkaline phosphatase reporter (RAW-Blue cells) with wild-type (WT), Δ*rgg2* (QS-OFF), or Δ*rgg3* (QS-ON) GAS. All cells were infected at an multiplicity of infection (MOI) of 10 unless otherwise indicated. Reporter activity (absorbance at 625 nm) is shown. (B) TNF-α and IL-6 production by RAW264.7 cells 8 h after infection. (C) LDH release as a measurement of macrophage cell death 8 h after infection. The percentage of dead cells was quantified using a 100% lysed cell control. (D) NF-κB activity after infecting RAW-Blue cells for 20 h with different MOIs of Δ*rgg2* (black bars) or Δ*rgg3* (blue bars) GAS. (E) TNF-α production by RAW264.7 cells 8 h after infection with different serotypes of GAS grown in the presence of revSHP (black bars) or SHP (blue bars). (F) TNF-α production by BMDM, BMDC, or THP-1 cells 8 (BMDM, BMDC) or 12 (THP-1) h after infection with GAS (strain NZ131) grown in the presence of revSHP (black bars) or SHP (blue bars). Means plus standard deviations (SD) are shown from a representative of three independent experiments conducted in triplicate. Statistical significance is indicated as follows: ***, *P* = 0.001; ****, *P* < 0.0001 by two-tailed unpaired *t* test (A and B) or ordinary one-way ANOVA with Tukey’s multiple-comparison test (C to E); ns, not significantly different.

Macrophages respond to GAS in part by activating NF-κB, a transcription factor capable of regulating expression of many proinflammatory cytokines. To determine whether the GAS QS state impacts macrophage responses, proinflammatory responses were measured following *in vitro* infections with wild-type (WT) (strain NZ131), Δ*rgg3* (QS-locked-ON), or Δ*rgg2* (QS-locked-OFF) GAS. Under standard culturing of GAS in a chemically defined medium (CDM) with glucose, the Rgg2/3 QS system remains silent in WT unless exogenous SHP (>1 nM) is supplemented (3), the endopeptidase PepO is inactivated (15, 16), or specialized conditions are met (i.e., mannose is substituted as the carbon source or if manganese or iron are depleted) (17). As expected, infection of macrophages with WT or Δ*rgg2* GAS grown in CDM resulted in macrophage stimulation, as indicated by activation of NF-κB and production of large amounts of tumor necrosis factor alpha (TNF-α) and interleukin 6 (IL-6) (Fig. 1A and B). Surprisingly, infection with the Δ*rgg3* mutant led to approximately 4-fold decreased NF-κB reporter activity and 20-fold decreased TNF-α and 25-fold decreased IL-6 production compared to infection with WT or Δ*rgg2* strain (Fig. 1A and B). To determine whether decreased activity was due to differences in macrophage cytotoxicity, extracellular release of the cytoplasmic enzyme lactate dehydrogenase (LDH) was measured as an indicator of plasma membrane damage. LDH release was minimal in response to WT and mutant GAS strains after 8 h of infection (Fig. 1C). Additionally, infecting macrophages with increasing multiplicities (MOIs) of the Δ*rgg2* mutant resulted in dose-dependent activation of NF-κB (Fig. 1D). Increasing the MOI of the Δ*rgg3* mutant, however, failed to increase NF-κB responses, and instead they remained attenuated even at the highest MOI tested. Whereas macrophages were stimulated by WT GAS grown in nonsupplemented CDM, macrophages infected with SHP-activated WT GAS led to attenuated TNF-α production similar to that of the genetically established QS-locked-ON mutant, the Δ*rgg3* mutant (Fig. 1E). Macrophage responses to WT GAS grown in the presence of the inactive peptide that contains the reversed sequence of SHP (revSHP) mimicked that of the genetically established QS-locked-OFF mutant, the Δ*rgg2* mutant.

Rgg2/3 QS is conserved across all sequenced serotypes of GAS. GAS serotypes are classified based on differences in the M protein, a surface protein, and a major virulence factor. The NZ131 strain (serotype M49) was used for a majority of this work because it is a highly transformable strain and excellent tool for genetic manipulation of GAS. To confirm that the immunomodulatory phenotype is not restricted to strain NZ131, macrophage TNF-α responses were measured following infection with other wild-type strains: HSC5 (M14), 5448 (M1), and MGAS315 (M3). These particular strains were chosen as representatives of diverse M types. Furthermore, we confirmed previously that Rgg2/3 activation leads to cell surface changes (lysozyme resistance and biofilm formation) in both HSC5 and MGAS315 strains (17). Strain 5448 was added as a representative M1T1 globally disseminated, clinically relevant strain. With the exception of MGAS315, activation of Rgg2/3 QS by the addition of exogenous SHP resulted in reduced macrophage TNF-α production in response to all other strains tested (Fig. 1E).

Curiously, minimal TNF-α was produced in response to strain MGAS315, even without the activation of QS. We hypothesized that this may be due to the strain’s high expression of capsule, a virulence factor that prevents adherence and phagocytosis (18). However, treatment of MGAS315 with hyaluronidase, an enzyme capable of cleaving the hyaluronic acid capsule, failed to increase the NF-κB response to Δ*rgg2* or Δ*rgg3* MGAS315 (see Fig. S1A, Δ*rgg2*-H and Δ*rgg3*-H, in the supplemental material).

10.1128/mBio.03400-20.3 FIG S1 QS does not alter bacterial adherence or internalization. (A) NF-κB activity after RAW-Blue cells were infected with the described mutant GAS strains in serotype MGAS315 or NZ131. Capsule digestion by hyaluronidase treatment of GAS is indicated by “-H” following the genotype. (B) TNF-α production 8 h after RAW264.7 cells were infected with the described GAS strains. For mixed infections, Δ*rgg2* GAS of serotype NZ131 was used to stimulate TNF-α production. (C) NF-κB activity after RAW-Blue cells were infected with hyaluronidase-treated (H) MGAS315 or untreated Δ*covR* mutant. For mixed infections, Δ*rgg2* GAS of serotype NZ131 was used to stimulate NF-κB activity. (D) GAS adherence to RAW264.7 cells after 30 min of infection. Adherence was calculated as the percentage of viable bacterial cells recovered compared to infection inoculum. (E) Gentamicin protection assay to determine the percentage of viable intracellular GAS compared to inoculum after infecting RAW264.7 cells with Δ*rgg2* or Δ*rgg3* GAS for 30 min and incubating with gentamicin (100 μg/ml) thereafter for the described amounts of time. Means ± SD are shown from a representative of two independent experiments conducted in triplicate. ***, *P* = 0.002, ****, *P* < 0.0001, by two-way ANOVA with Sidak’s multiple-comparison test (A) and ordinary one-way ANOVA with Tukey’s multiple-comparison test (B). Download FIG S1, TIF file, 1.6 MB. Copyright © 2021 Rahbari et al. 2021 Rahbari et al. https://creativecommons.org/licenses/by/4.0/ This content is distributed under the terms of the Creative Commons Attribution 4.0 International license.

To determine whether the differential cytokine response is restricted to RAW264.7 macrophages, the effect of QS activation on cytokine production was also examined following *in vitro* infection of primary mouse bone marrow-derived macrophages (BMDM) and dendritic cells (BMDC), as well as phorbol myristate acetate (PMA)-differentiated THP-1 human monocyte cells. TNF-α production by BMDM, BMDC, and THP-1 cells was attenuated in response to the Δ*rgg3* mutant, consistent with responses seen in RAW264.7 cells (Fig. 1F). These data suggest that the Δ*rgg3* mutant is able to manipulate cytokine production in multiple innate immune cell types, including human macrophages, indicating the potential relevance and applicability of interfering with QS as a therapeutic strategy.

### QS-ON GAS actively suppresses macrophage inflammatory responses.

**FIG 2:**
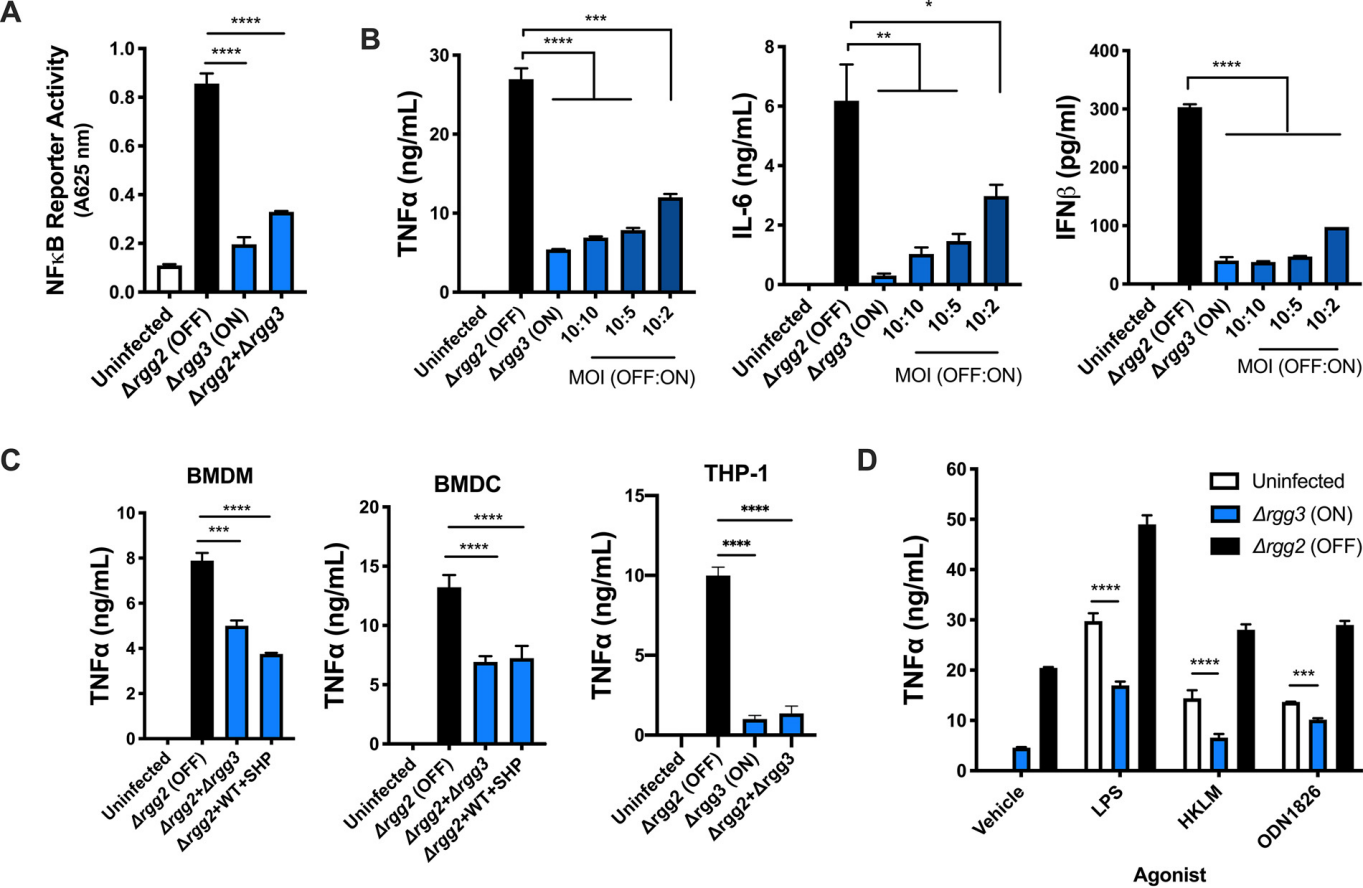
QS-ON GAS actively suppresses inflammatory responses. (A) NF-κB activity after infecting RAW-Blue cells with Δ*rgg2* or Δ*rgg3* GAS or both. (B) TNF-α, IL-6, and IFN-β production after RAW264.7 cells were infected with the described MOIs of Δ*rgg2* and Δ*rgg3* GAS. (C) TNF-α production by BMDM, BMDC, and THP-1 cells 8 (BMDM, BMDC) or 12 (THP-1) h after infection at an MOI of 10 of Δ*rgg2* GAS or coinfected with an MOI of 10 of each Δ*rgg2* and Δ*rgg3* GAS or Δ*rgg2* and WT GAS grown in the presence of SHP. (D) TNF-α production after RAW264.7 cells were coincubated with TLR agonists and Δ*rgg3* or Δ*rgg2* GAS. LPS, lipopolysaccharide (TLR4); HKLM, heat-killed Listeria monocytogenes (TLR2); ODN1826, CpG oligodeoxynucleotide (TLR9). Means plus SD are shown from a representative of two (D) or three (A to C) independent experiments conducted in triplicate. *, *P* < 0.05; **, *P* < 0.005; ***, *P* < 0.001; ****, *P* < 0.0001, by ordinary one-way ANOVA with Tukey’s multiple-comparison test (A to C) or two-way ANOVA with Sidak’s multiple-comparison test (D).

To distinguish between the possibilities that QS-activated GAS either passively evades detection (i.e., hides) or actively downregulates (i.e., suppresses) inflammatory responses, macrophages were infected with mixed populations of Δ*rgg3* and Δ*rgg2* mutants, and NF-κB activity and cytokine production were measured. Following mixed infection with both Δ*rgg3* and Δ*rgg2* mutants, NF-κB activity remained low and was similar to that of Δ*rgg3* single infection (Fig. 2A). Decreasing the ratio of the Δ*rgg3* to Δ*rgg2* mutant (by decreasing the MOI of the Δ*rgg3* mutant) in mixed infections led to a dose-dependent increase in production of all cytokines tested (TNF-α, IL-6, and interferon beta [IFN-β]). Surprisingly, cytokine production remained attenuated even when the Δ*rgg3* mutant was outnumbered 5 to 1 by the Δ*rgg2* mutant (Fig. 2B). TNF-α production by primary mouse BMDM and BMDC and human THP-1 cells was also attenuated following mixed infections with equal MOIs of Δ*rgg2* and either Δ*rgg3* or WT strains grown in the presence of SHP (Fig. 2C). Together, these data suggest that QS enables GAS to suppress macrophage cytokine responses.

Because infecting macrophages with MGAS315 elicited low TNF-α production (Fig. 1E), we wondered whether this strain could suppress macrophage responses irrespective of the QS state. MGAS315 strains lacking *rgg3* or *rgg2* were used to determine the effect on cytokine production in mixed infections; the QS-OFF strain (Δ*rgg2*-NZ131 background) was used to elicit TNF-α responses. Similar to WT MGAS315 (Fig. 1E), single infections with either the Δ*rgg2* or Δ*rgg3* mutant resulted in undetectable TNF-α production (data not shown). Mixed infections resulted in minor amounts of suppression, independent of the QS state (Fig. S1B). Again, we wondered if high levels of capsule on MGAS315 prohibited the suppressive capacity in this strain and therefore treated cultures with hyaluronidase, confirming the loss of capsule by the ability to pellet cells after brief centrifugation. However, hyaluronidase treatment of strain MGAS315 did not potentiate QS-ON GAS to suppress (Fig. S1C), indicating that MGAS315 is unique among strains tested in that it cannot utilize QS to suppress NF-κB or TNF-α responses.

The specific pathogen-associated molecular patterns (PAMPs) responsible for inducing proinflammatory responses to GAS are incompletely described; however, it has been well established that both the MyD88−NF-κB pathway and the type I IFN pathway are required for clearance (12, 14). We wondered whether QS would enable GAS to suppress stimulation by TLR agonists. TLR agonist-stimulated macrophages produced significantly larger amounts of TNF-α when applied simultaneously with the Δ*rgg2* mutant (Fig. 2D). However, agonist treatment together with Δ*rgg3* infection resulted in decreased TNF-α production in response to lipopolysaccharide (LPS; TLR4), heat-killed Listeria monocytogenes (HKLM; TLR2), and CpG oligodeoxynucleotide (ODN1826; TLR9). Together, these data suggest that GAS suppresses NF-κB-mediated proinflammatory responses downstream of TLR stimulation.

### Suppression requires contact with live QS-ON GAS.

**FIG 3:**
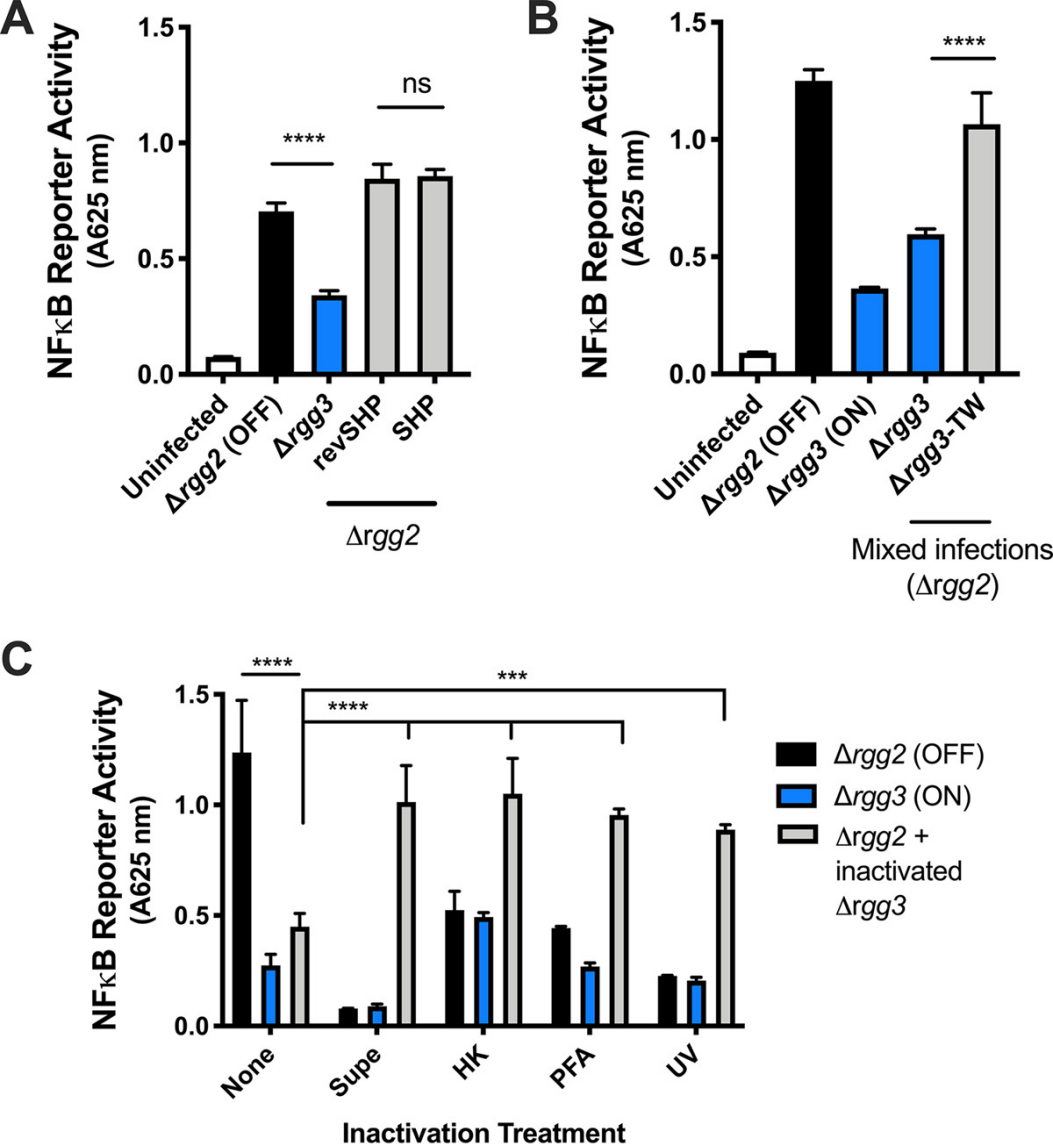
Suppression requires contact with live QS-ON GAS. (A) NF-κB activity after stimulating RAW-Blue cells with an MOI of 10 of Δ*rgg2* GAS and treating with 200 nM SHP or revSHP or an MOI of 10 of Δ*rgg3* GAS. (B) NF-κB activity after infecting RAW-Blue cells directly with the described strains or with Δ*rgg3* GAS separated by a transwell with 0.4-μm pores (TW). (C) NF-κB activity after infecting RAW-Blue cells with live Δ*rgg2* GAS and/or GAS that was inactivated with heat treatment (HK), short wave UV light treatment (UV), or 4% paraformaldehyde (PFA), or OD-normalized sterile filtered supernatants (Supe). Means plus SD are shown from a representative of three independent experiments conducted in triplicate. ***, *P* < 0.001; ****, *P* < 0.0001 by two-way ANOVA with Sidak’s multiple-comparison test (A and B) by ordinary one-way ANOVA with Tukey’s multiple-comparison test (C).

To localize the factor(s) responsible for QS-regulated cytokine modulation, the ability of secreted/extracellular components and surface-associated components to alter NF-κB activity was tested. In other pathogenic bacteria, QS molecules themselves have been shown to possess immunomodulatory properties toward host cells. For example, treatment of epithelial cells with QS molecules produced by Pseudomonas aeruginosa (i.e., homoserine lactones, quinolones, and phenazines) results in differential regulation of cytokine expression via sensing by the host receptor AhR (19). Macrophage treatment with the Rgg2/3 QS molecule SHP failed to increase NF-κB activity compared to untreated controls. Therefore, we tested whether addition of SHP to macrophages infected by the Δ*rgg2* mutant was sufficient to attenuate NF-κB activity. We saw that addition of 200 nM either SHP or revSHP to macrophages was unable to suppress NF-κB activity (Fig. 3A). These data suggest that although SHP signals induce Rgg2/3 signaling, they are not directly responsible for the observed immunomodulatory effect.

To determine whether other secreted factors could enable QS-ON GAS to suppress NF-κB activity, macrophages were directly stimulated with Δ*rgg2* GAS, and Δ*rgg3* GAS was added to macrophages either directly or separated by a 0.4-μm-pore transwell membrane (TW). This pore size prohibits Δ*rgg3* GAS from directly contacting macrophages while simultaneously allowing secreted factors to pass through the membrane. We determined that when Δ*rgg3* GAS was added above the TW (Δ*rgg3-*TW), it was unable to suppress NF-κB activity (Fig. 3B, gray bar). This indicated that macrophage suppression is a contact-dependent phenomenon. Furthermore, sterile filtered supernatants from Δ*rgg3* cultures were added to macrophages infected with Δ*rgg2* GAS and failed to suppress NF-κB activity, further indicating that GAS culture supernatants lack the factor responsible for suppressing macrophage responses (Fig. 3C).

Our previous work illustrated that cell surface properties of GAS are altered via Rgg2/3 QS (3, 5, 17). To examine whether these surface changes include alterations of the repertoire or display of antigens, macrophages were infected with GAS inactivated by different methods, including heat (heat killed [HK]), short wave UV light (UV), and 4% paraformaldehyde (PFA) treatment. While heat has been shown to alter cell wall peptidoglycans and denature proteins, UV treatment damages DNA and RNA and is less likely to interfere with cell surface properties. PFA treatment cross-links amines in proteins and other structures within the cell. We posited that if QS-induced surface changes are responsible for altered macrophage responses, infection with inactivated GAS would similarly result in differential inflammatory responses. After each inactivation treatment, bacteria were plated for enumeration to confirm loss of viability, and in each case >99.9% of cells were nonrecoverable. Macrophages infected with inactivated GAS had lower NF-κB activity compared to those infected with live GAS, and this was consistent across all treatments (Fig. 3C). Inactivation of GAS with heat and UV eliminated the QS-dependent differences in NF-κB activity (Fig. 3C). Furthermore, inactivation of Δ*rgg3* GAS by any treatment eliminated its ability to attenuate NF-κB activity when added to cells stimulated with live, metabolically active Δ*rgg2* GAS (Fig. 3C). This suggests that alterations in surface properties are unlikely responsible for the differences in macrophage responses to Δ*rgg3* GAS, though it is possible that these inactivation methods may have interfered with the unknown ligand responsible for immunosuppression. Cumulatively, these data indicate that immunomodulation by QS-ON GAS requires live bacteria and direct contact between the pathogen and host cells.

### QS does not alter bacterial adherence or internalization.

GAS initially interacts with macrophages through bacterial adherence and internalization. Though initially thought to be an exclusively extracellular pathogen, much evidence has illustrated that GAS can also invade and even replicate intracellularly (20, 21). We found that immunosuppression requires contact, as it does not occur when QS-ON GAS is separated from macrophages by a transwell insert (Fig. 3B). To determine whether adherence to macrophages is altered by QS activity, GAS was plated for CFU enumeration after infection and compared to the initial inoculum. The Δ*covR* mutant (NZ131) was utilized as a negative control due to its high level of capsule (an antiphagocytic and anti-adherence factor). Although the Δ*covR* mutant was a poor binder, the ability of WT, Δ*rgg2*, and Δ*rgg3* strains to adhere to macrophages after 30 min was clearly apparent but not significantly different from one another (Fig. S1D) (22).

Macrophages can sense extracellular GAS through various cell surface receptors, but they can also recognize intracellular GAS through cytosolic and endosomal receptors. To determine whether the QS state results in altered internalization or intracellular survival in macrophages, viable GAS was measured over time after gentamicin treatment. Both Δ*rgg2* and Δ*rgg3* bacteria were internalized to similar levels, and at hourly intervals following gentamicin treatment, we found that the numbers of viable bacteria were reduced at similar rates. Thus, the ability of Δ*rgg3* bacteria to suppress macrophage activity is not a function of an altered ability to internalize or survive phagocytosis (Fig. S1E).

### A QS-regulated operon is required for cytokine suppression.

**FIG 4:**
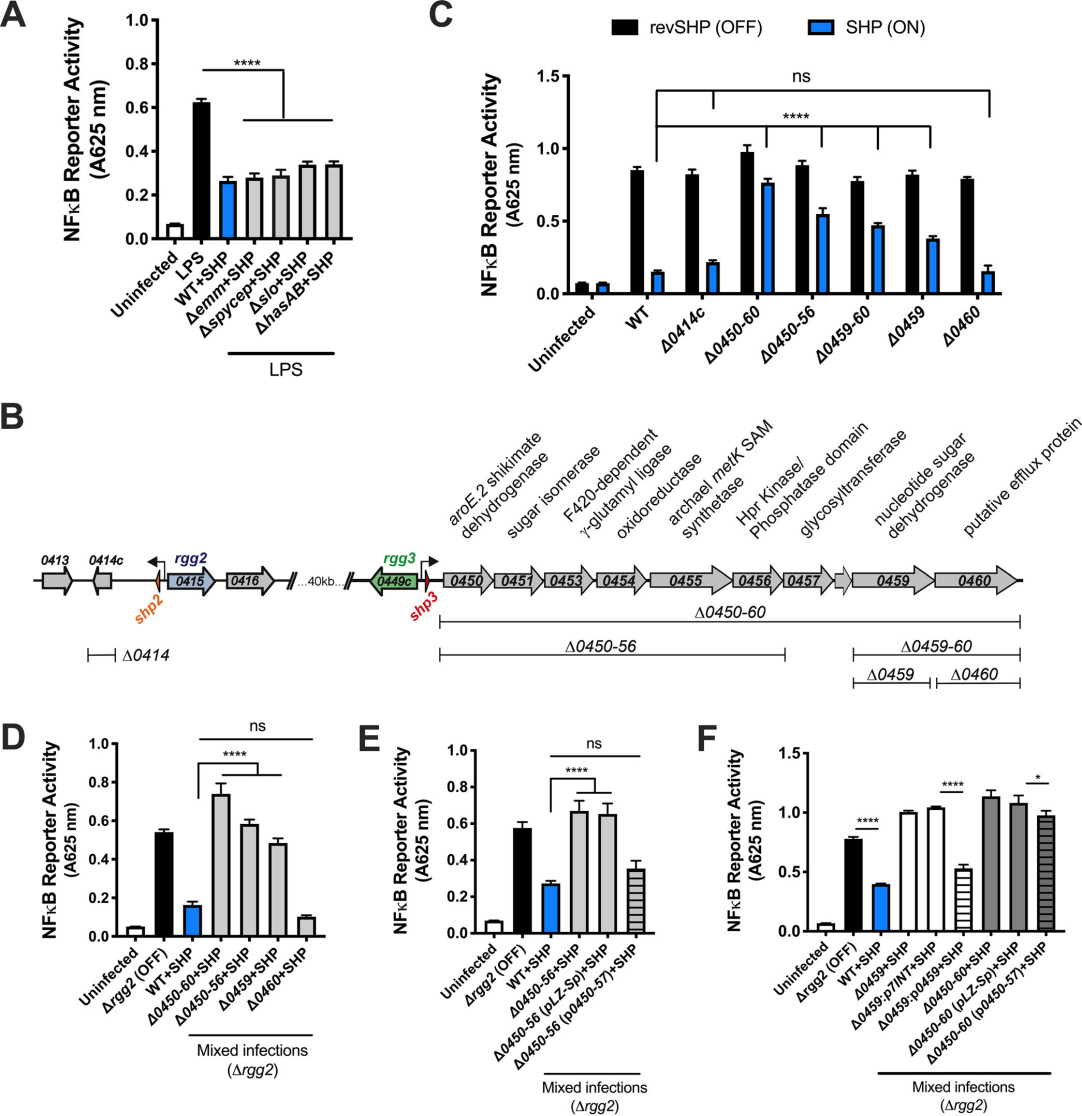
A QS-regulated biosynthetic gene cluster (BGC) is required for cytokine suppression. (A) NF-κB activity of RAW-Blue cells infected with GAS isogenic mutants lacking various virulence factor genes and grown in the presence of SHP. Following 30 min of infection, macrophages were stimulated with LPS. (B) Diagram depicting the QS-regulated genetic programs in GAS (strain NZ131), putative functions of the genes, and the isogenic mutants used in subsequent assays. (C) NF-κB activity after RAW-Blue cells were infected with the described GAS genetic mutant strains grown in the presence of either inactive reverse SHP (revSHP) or active SHP. (D) NF-κB activity after RAW-Blue cells were coinfected with Δ*rgg2* GAS at an MOI of 10 of the described BGC mutants grown in the presence of SHP. (E and F) NF-κB activity after RAW-Blue cells were stimulated with Δ*rgg2* GAS and infected with BGC mutants and complementation strains. All GAS strains were grown in the presence of SHP to induce Rgg2/3 QS activity. Means plus SD are shown from a representative of three independent experiments conducted in triplicate. *, *P* < 0.05; ****, *P* < 0.0001, by ordinary one-way ANOVA with Tukey’s multiple-comparison test (A and D to F) or two-way ANOVA with Sidak’s multiple-comparison test (panel C). ns, not significantly different.

Several virulence factors have been described as important in manipulating immune responses to GAS, including streptolysin O (SLO), S. pyogenes cell envelope protease (SpyCEP), M protein, and capsule, all of which interact with the bacterial surface (23, 24). To test whether any of these components are involved in QS-dependent immunosuppression, we stimulated the Rgg2/3 system by addition of SHP pheromone in isogenic strains lacking SLO (Δ*slo*), capsule (Δ*hasAB*), M protein (Δ*emm*), or SpyCEP (Δ*spycep*). When QS was stimulated, each mutant maintained its capacity to suppress NF-κB activity in LPS-activated macrophages, indicating that these virulence factors are not involved in the QS-dependent phenotype (Fig. 4A).

The major genetic targets of the Rgg2/3 QS pathway include (i) *spy49_0414c*, encoding the protein StcA, and (ii) *spy49_0450-0460*, encoding a putative biosynthetic gene cluster (BGC) (Fig. 4B) (3). We previously reported that activation of Rgg2/3 QS mediates production of biofilms and resistance to the host antimicrobial factor lysozyme, two phenotypes that rely on surface modifications and were shown to require QS regulation of StcA (3, 5). We wondered whether regulation of StcA also enabled suppression of inflammatory pathways in innate immune cells. Macrophages were infected with either Δ*0414c* or Δ*0450-0460* GAS, each grown in the presence of SHP (QS-ON) or inactive revSHP (QS-OFF), and NF-κB activity was evaluated. The outcomes of the infections showed that while *spy49_0414c* was not required for QS-regulated attenuation of NF-κB activity, *spy49_0450-0460* was necessary (Fig. 4C). Further interrogation using isogenic mutants of various genes within this operon determined that QS-ON GAS lacking either the first six genes (*spy49_0450-0456*) or the second to last gene, *spy49_0459*, failed to attenuate NF-κB activity following single infections (Fig. 4C). Interestingly, infection with QS-ON GAS lacking only *spy49_0460* attenuated NF-κB activity to similar levels as infection with the WT control (Fig. 4C). Consistent with this observation, mixed infections with QS-OFF GAS and WT or BGC mutants grown in the presence of SHP pheromone (QS-ON) demonstrated that BGC mutants significantly restored the NF-κB response, suggesting these genes are involved in suppression (Fig. 4D). Expressing *spy49_0450-0457* from an episomal plasmid, or *spy49_0459* in single copy from a phage integration site in the chromosome, as a means to complement corresponding gene deletions at the native location was able to restore the suppressive capacity of GAS (Fig. 4E and F). However, complementation of Δ*0450-0460* with *spy49_0450-0457* failed to fully restore suppression, suggesting that all genes in the BGC (with the exception of *spy49_0460*) are needed for optimal suppressive activity (Fig. 4F). Together, these data suggest that the previously described surface alterations mediated by *stcA* are not responsible for macrophage suppression by QS-ON GAS and instead genes in the BGC are involved.

### Rgg2/3 QS can be targeted pharmacologically to restore immunity.

**FIG 5:**
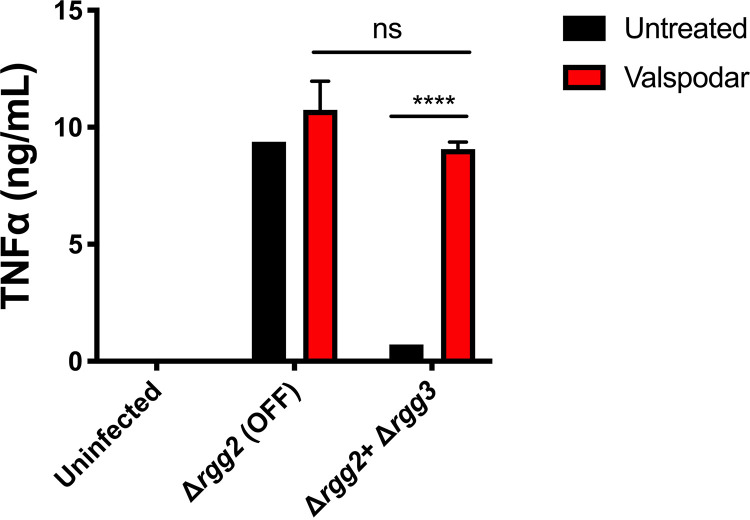
Rgg2/3 QS can be targeted pharmacologically to restore immunity. TNF-α production after RAW264.7 cells were infected with GAS that was treated with the QS inhibitor valspodar (10 μM) or not treated with valspodar. Means plus SD are shown from a representative of three independent experiments conducted in triplicate. ****, *P* < 0.0001, by two-way ANOVA with Tukey’s multiple-comparison test.

The ability of QS-activated GAS to suppress host cytokine responses could be a new potential mechanism to target *in vivo* as an antivirulence treatment strategy. Previous screening of a library of FDA-approved drugs identified cyclosporine A (CsA) as an inhibitor of Rgg2/3 QS signaling. The CsA analog valspodar, which lacks immunosuppressive effects, was also found to inhibit signaling (25). To determine whether pharmacologically inhibiting QS could prevent immunosuppression, Δ*rgg3* and Δ*rgg2* cultures were treated with 10 μM valspodar and then applied to macrophages. Compared with untreated Δ*rgg3* GAS, valspodar-treated GAS was no longer able to suppress cytokine production in macrophages (Fig. 5). These data indicate that blocking QS signaling can restore macrophage responses to GAS and eliminate their ability to suppress cytokine production.

## DISCUSSION

This study presents a novel phenomenon whereby GAS utilizes an intercellular regulatory system to actively suppress NF-κB activity and proinflammatory cytokine production in macrophages and dendritic cells. Bacteria incapable of responding to pheromone signals (QS-OFF mutants) lacked the ability to suppress immune cell responses. QS-ON GAS that were inactivated by heat treatment, fixation, or UV irradiation also lost their ability to suppress inflammatory responses. This indicates that suppression is an active process occurring at the interface of GAS and macrophages rather than presentation of a stable surface moiety by GAS. Cell-free culture supernatants also failed to suppress inflammatory responses, further supporting the notion that a secreted factor is not released into the surrounding milieu. Separation of bacteria from stimulated macrophages by a transwell membrane also abolished suppression capacity of QS-ON GAS. Together, these data support a model whereby GAS contacts innate immune cells and, when QS signaling is active, presents or delivers a factor that interrupts host signaling pathways.

The Rgg2/3 QS system directly regulates two operons, each encoding an *shp* gene and additional coding sequences: *spy49_0414c* (*stcA*) and *spy49_0450-0460*, a putative biosynthetic gene cluster. Here, we were able to link immune cell suppression activity to expression of *spy49_0450-0460*; deletion of the operon abolished suppression. This locus is conserved across all sequenced GAS genomes and is comprised of 10 genes coexpressed from the *shp3* promoter. They encode various enzymes involved in amino acid biosynthesis, sugar metabolism, and capsule biogenesis (Fig. 4B). The operon was found to be repressed by the metal-dependent regulator MtsR, and some of the genes were identified in screens for those involved in invasive infection and survival in blood (26–28). Yet, little remains known about this putative biosynthetic gene cluster, and similar gene sets are seen in only a few other instances in Streptococcus porcinus, Streptococcus pseudoporcinus, Salinispora tropica, and Bacillus thuringiensis. Curiously, in B. thuringiensis, the homologous gene cluster is implicated in the biosynthesis of a secreted toxin called thuringensin, a nucleoside analog predicted to have cytotoxic activity (29). We observed no cytotoxic impact to macrophages incubated with QS-ON or QS-OFF cells (Fig. 1C); however, we hypothesize that the product of the *spy49_0450-0460* gene cluster could be a bioactive molecule produced in response to and directed at macrophages. The last gene of the operon, *spy49_0460* (*mefE*), encodes a putative efflux protein. The presence of this gene in the operon is consistent with the notion that it aids in delivery of a molecule. Surprisingly, however, deletion of *mefE* did not affect QS-mediated cytokine suppression in macrophages (Fig. 4D), refuting its potential involvement in the observed activity, unless a redundant pathway is capable of releasing a factor.

Bacteria use diverse strategies to modulate host responses. We hypothesize that GAS produces a QS-regulated factor that can manipulate host signaling pathways and subsequently shape cytokine responses. Upon TLR activation, a cascade of phosphorylation and ubiquitination events leads to degradation of the inhibitor IκB, allowing NF-κB translocation to the nucleus where it regulates transcription. Here, we demonstrate that GAS suppresses stimulation at a step downstream of TLR activation and upstream of NF-κB transcriptional induction. Several examples of NF-κB pathway disruptors have been reported for Gram-negative pathogens and include *Yersinia* YopJ, Escherichia coli Nle proteins, and *Shigella* Osp proteins (30). Fewer examples have been reported for Gram-positive pathogens which largely lack specialized secretion systems used to deliver factors directly to host cells. We found that QS-induced suppression of macrophages required contact with live GAS and was not found in supernatants (Fig. 3). This suggests that the factor is not released from bacteria into extracellular spaces, but instead it might be delivered by a mechanism allowing translocation across the host membrane, such as that seen in type III secretion systems (T3SS). In GAS, delivery of the virulence factor NAD-glycohydrolase (Nga) has been shown to occur in a mechanism functionally equivalent to T3SS but requiring the pore-forming toxin SLO for delivery (31, 32). We induced the Rgg2/3 system in a strain lacking SLO (Δ*slo*) and found it maintained its capacity to suppress NF-κB (Fig. 4A). Thus, if Rgg2/3-dependent immune suppression involves a translocated factor, effector delivery may involve uncharacterized delivery mechanisms.

The ability of Staphylococcus aureus, another Gram-positive pathogen, to actively interfere with host signaling components does not necessitate translocation of factors into host cells. For example, staphylococcal superantigen-like protein (SSL3) binds TLR2 and blocks ligand binding and TLR heterodimerization, thereby inhibiting macrophage stimulation (33, 34). Another protein, TirS, blocks TLR-dependent NF-κB activation by mimicking and interfering with host Toll−interleukin 1 receptor (TIR)-containing adaptor proteins (like MyD88), and unlike SSL3, presumably must enter the host cell cytoplasm in order to function (35). How TirS gains access to the host cytosol is unresolved.

Although many GAS virulence factors facilitate immune evasion by various mechanisms, none have been reported to suppress NF-κB responses. Multiple virulence factors in GAS can suppress cytokines posttranslationally. For example, SLO induces host degradation of IL-1β via ubiquitination, and SpyCEP directly degrades IL-8 via proteolytic cleavage (36, 37). Here, we describe a distinct mechanism of suppression upstream of these pathways. Interestingly, inoculation of macrophages with MGAS315 did not induce cytokine responses regardless of the QS state, but it failed to exhibit immunosuppressive capacity (Fig. 1E; see also Fig. S1C in the supplemental material). Further exploration of the differences between the MGAS315 strain and the others could help shed light on the bacterial factors responsible for the differences in macrophage responses. We suspect that differences in capsule, which prevents adherence to host cells, are involved because direct contact was required for suppression (Fig. 3). Though hyaluronidase cleavage of capsule did not potentiate QS-ON MGAS315 to suppress, a capsule mutant should be tested to verify whether this strain lacks the QS-regulated components responsible for immunosuppression. Interestingly, deleting capsule genes in strain NZ131 did not improve suppression compared to the WT, suggesting there may be redundant factors involved.

Rgg2/3 QS is activated in response to environmental conditions found in host mucosal environments, including metal depletion and mannose utilization (17). It remains unclear what benefits are afforded by the additional layer of intercellular communication signaling. We recently demonstrated the importance of Rgg2/3 QS activation *in vivo* for nasopharyngeal colonization (38). Whether Rgg2/3-mediated immunosuppression was a contributing factor in the observed increased colonization rates of *rgg3* mutants (QS-ON) was not a component of these studies, but blocking NF-κB signaling would likely diminish the efficacy of host strategies for bacterial eradication, including production of antimicrobial factors (lysozyme, cationic peptides), induction of autophagic responses, and recruitment of additional phagocytic and cytotoxic immune cells. Incorporating cell-cell signaling under threatening conditions likely reinforces bacterial compliance to engage defense mechanisms that improve probabilities of survival if immune responses are blunted.

As we gain deeper understanding of how QS systems are used by bacteria to enhance fitness attributes in the context of host colonization and/or infection, the more promising it becomes to target signaling activities pharmacologically as an alternative or supplementary therapeutic approach to antibiotics. We previously determined that the FDA-approved drugs cyclosporine and valspodar have specific inhibitory activities against Rgg2/3 QS (25). We are encouraged by results shown here that blocking QS activity with valspodar abolished its cytokine attenuation effects and restored macrophage inflammatory responses to GAS *in vitro*. Testing methodologies that incorporate anti-QS therapeutics remains a compelling strategy that could bolster the host’s intended immune response and diminish infections by this global pathogen.

## MATERIALS AND METHODS

### Bacterial strains.

S. pyogenes (group A streptococcus [GAS]) strain NZ131 was used as the parental strain for genetic mutants (constructed as described below). Other GAS strains tested included HSC5, 5448, and MGAS315. GAS were routinely grown without shaking at 37°C in Todd-Hewitt broth with 0.2% yeast extract (THY) or on agar plates, or in a chemically defined medium (CDM) containing 1% (wt/vol) glucose (39). To induce Rgg2/3 activation, 100 nM synthetic SHP pheromone or inactive reverse SHP (revSHP) was added to S. pyogenes cultures at an optical density at 600 nm (OD_600_) of ∼0.1. When appropriate, antibiotics were added at the following concentrations: chloramphenicol (Cm), 3 μg/ml; erythromycin (Erm), 0.5 μg/ml; kanamycin (Kan), 150 μg/ml; spectinomycin (Sp), 100 μg/ml. Starter cultures were utilized to minimize differences in lag phase and were prepared as follows. GAS strains of interest were streaked on THY plates containing the appropriate antibiotics. Single clones were isolated and inoculated into THY containing the appropriate antibiotics. After incubation overnight at 37°C, the cultures were diluted 1:20 into CDM and grown at 37°C to mid-exponential phase (OD_600_, 0.5 to 0.8). Last, glycerol was added to a final concentration of 20%, and single-use aliquots were stored at −80°C.

### Construction of mutant strains and complementation plasmids.

All bacterial strains used in this study are described in Table S1 in the supplemental material. Primers used to construct plasmids for gene deletion or complementation are listed in Table S2. All cloning was done using laboratory E. coli cloning strains such as NEB-5a (New England Biolabs) or BH10c (40), with antibiotics added at the following concentrations: Cm, 10 μg/ml; Erm, 500 μg/ml; Sp, 100 μg/ml. To construct gene deletions, sequences flanking the gene of interest were amplified by PCR and ligated into a temperature-sensitive plasmid, pFED760, by restriction enzyme digestion or Gibson assembly. When necessary, antibiotic resistance markers for kanamycin (*aphA3*) or chloramphenicol (*cat*) were cloned between the upstream and downstream flanking regions to generate plasmids for selective allelic replacement. Deletion vectors were electroporated into strain NZ131, and a two-step temperature-dependent selection process was used to isolate mutants of interest. Briefly, cells containing each deletion construct were grown at the permissive temperature, then shifted to 37°C, and plated on the appropriate antibiotic to select for bacteria in which the plasmid had integrated at one of the flanking regions. After confirmation of plasmid integration by PCR, cells were grown for ∼50 generations at the permissive temperature to allow the plasmid to recombine out, and loss of antibiotic resistance was used to identify the desired mutants. Genotypes were confirmed by PCR. To construct complementation plasmids for BGC mutants, the genomic region encompassing *spy49_0450-0457* and its native promoter were amplified by PCR and cloned into a multicopy vector (pLZ12-Sp). For complementation of *spy49_0459*, the gene was amplified by PCR and cloned downstream of sequence containing ∼70 bp of the *shp3* promoter in an integrating shuttle vector (p7INT/pJC420). Complementation plasmids were confirmed by sequencing.

10.1128/mBio.03400-20.1 TABLE S1 Strains and plasmids used in this study. Download Table S1, DOCX file, 0.03 MB. Copyright © 2021 Rahbari et al. 2021 Rahbari et al. https://creativecommons.org/licenses/by/4.0/ This content is distributed under the terms of the Creative Commons Attribution 4.0 International license.

10.1128/mBio.03400-20.2 TABLE S2 Primers used in this study. Download Table S2, DOCX file, 0.02 MB. Copyright © 2021 Rahbari et al. 2021 Rahbari et al. https://creativecommons.org/licenses/by/4.0/ This content is distributed under the terms of the Creative Commons Attribution 4.0 International license.

### Cell lines.

NF-κB reporter macrophages (RAW-Blue, InvivoGen) were cultured in Dulbecco modified Eagle medium (DMEM) (Gibco) supplemented with 10% fetal bovine serum (FBS) (Gemini, BenchMark), penicillin-streptomycin (P-S) (Corning), and zeocin (InvivoGen), and RAW264.7 macrophages were maintained in RPMI 1640 (Corning) supplemented with 10% FBS and P-S. THP-1 cells were cultured in RPMI 1640 supplemented with 10% FBS, P-S, and 0.05 mM 2-mercaptoethanol. All cell lines were maintained and passaged at 37°C and 5% CO_2_.

### Primary cell culture.

BMDM and BMDC were generated from bone marrow precursor cells extracted from the femurs and tibiae of 6- to 8-week-old male or female C57BL/6 mice (Charles River Laboratories).

### Synthetic pheromone peptides.

Synthetic peptides of 95% purity were purchased from NeoPeptide (Cambridge, MA), reconstituted as 1 mM stocks in dimethyl sulfoxide (DMSO), and stored at −80°C. Dilutions for working stocks were made in DMSO and stored at −20°C. The SHP3-C8 peptide (SHP) sequence is DIIIIVGG, and the reverse peptide (rev-SHP) sequence is GGVIIIID.

### *In vitro* infections.

A total of 2.5 × 10^5^ macrophages were seeded into 24-well tissue culture treated plates (Corning) in 500 μl of medium supplemented with penicillin-streptomycin the day before infection. The following day, medium was aspirated and replaced with medium without antibiotics. GAS was grown from starter cultures in CDM to an OD_600_ of 0.5 (∼2 to 3 h), then normalized based on OD, washed in phosphate-buffered saline (PBS), and added to macrophages. Unless otherwise noted, macrophages were inoculated with GAS at a multiplicity of infection (MOI) of 10:1. Cells were immediately centrifuged at 200 × *g* for 5 min to equilibrate infection and then incubated at 37°C with 5% CO_2_. After 30 min, extracellular bacteria were killed by replacing the medium with medium containing 100 μg/ml gentamicin (Gibco). Where applicable, cells were treated with the following TLR agonists from InvivoGen: lipopolysaccharide (LPS) (100 ng/ml), heat-killed Listeria monocytogenes (HKLM) (1 × 10^8^ cells), or CpG oligodeoxynucleotides (ODN1826) (1 μM). For experiments with attenuated GAS, bacteria were killed with heat (65°C), UV (short wave), or 4% PFA for 60 min. For experiments using GAS supernatants, cells were grown to an OD_600_ of ∼0.5 and pelleted, and the supernatants were subsequently filtered (0.2 μm) and normalized based on OD. For transwell experiments, 0.4-μm pores (Corning) were used. For hyaluronidase treatment of GAS, 1 × 10^8^ CFU GAS was collected at mid-exponential phase, washed, resuspended in 1 ml of PBS, and treated with 10 U/ml hyaluronidase (Sigma) for 10 min at 37°C. Removal of capsule was confirmed visually by morphological change in the cell pellet after centrifugation. Cells were washed with PBS to remove remaining enzyme and used for infection immediately after.

### Cell viability assay.

Cytotoxicity of macrophages after 8 h of infection was quantified by measuring the release of the enzyme lactate dehydrogenase in cell supernatants using the CytoTox 96 nonradioactive cytotoxicity assay (Promega) according to the manufacturer’s instructions. Percentages were determined by comparing samples with a 100% lysis control.

### NF-κB activation assay.

RAW-Blue macrophages (InvivoGen) with chromosomally integrated NF-κB inducible secreted embryonic alkaline phosphatase (SEAP) reporter construct were used to measure activation of NF-κB. Cells were maintained in DMEM (Gibco) supplemented with 10% heat-inactivated FBS (Gemini), penicillin-streptomycin (Corning), and 200 μg/ml zeocin (InvivoGen) in tissue culture treated T25 flasks (Greiner Bio-One). Cells were grown to 60 to 70% confluence, washed with PBS, and then passaged using 0.05% trypsin−0.53 mM EDTA (Corning) to dissociate them from the flasks. Reporter macrophages were counted and seeded the day prior to the experiment in flat bottom 96-well tissue culture treated plates (Corning) at 5 × 10^4^ cells/well. The following day, GAS was added to the wells at a multiplicity of infection of 10:1, and cells were centrifuged for 5 min to equilibrate infection. After a 30-min incubation at 37°C, the medium was removed and replaced with medium containing 100 μg/ml gentamicin. After 18 h, RAW-Blue cell-free supernatants were collected, and 50 μl was mixed with 150 μl of the QUANTI-Blue substrate (InvivoGen) and incubated at 37°C for 45 min in a flat bottom 96-well plate. Color change was quantified by measuring absorbance at 625 nm using a Synergy HTX microplate reader (BioTek).

### Generation of bone marrow-derived macrophages and dendritic cells.

Primary murine bone marrow-derived macrophages (BMDM) and dendritic cells (BMDC) were differentiated from bone marrow cells collected from the femurs and tibiae of 6- to 8-week-old male C57BL/6 mice (Charles River Laboratories). For macrophage differentiation, bone marrow cells were plated in 100 × 15 mm petri dishes in 10 ml DMEM supplemented with 10% FBS, 1 mM HEPES buffer (Gibco), 1× penicillin-streptomycin (Corning), and 10 ng/ml macrophage colony-stimulating factor (M-CSF) (BioLegend) and incubated at 37°C plus 5% CO_2_. On day 3, medium was removed, and cells were given 10 ml fresh medium. On day 7, the cells were washed gently with PBS and incubated with trypsin for 5 min at 37°C. Cells were then removed by gentle pipetting, counted, and plated at 2.5 × 10^5^ cells per well on 24-well plates or 5 × 10^4^ cells per well on 96-well plates overnight and infected on day 8.

For dendritic cell differentiation, red blood cells were lysed using RBC Lysis Buffer (Tonbo), and following a centrifugation step, cells were counted and plated on six-well plates in RPMI plus 5% FBS plus 20 ng/ml granulocyte-macrophage colony-stimulating factor (GM-CSF) (BioLegend). Cells were maintained at 37°C 5% CO_2_ and supplemented on days 3, 6, and 8. On day 10, cells were supplemented with medium containing 12.5 ng/ml GM-CSF. Cells were collected on day 11 and infected on day 12.

The purity of BMDMs and BMDCs was measured by cell surface staining and flow cytometry. A total of 2.5 × 10^5^ cells were transferred to an Eppendorf tube and resuspended in fluorescence-activated cell sorting (FACS) buffer (PBS plus 1% bovine serum albumin [BSA]). Prior to staining, cells were treated with anti-CD16/CD32 (BioLegend) for 15 min to block Fc receptors. Cells were then stained for 30 min on ice with allophycocyanin-Fire 750-labeled anti-CD11b (anti-CD11b-APC/Fire 750) and phycoerythrin-labeled anti-F4/80 (anti-F4/80-PE) for macrophages, or Alexa Fluor 647-labeled anti-CD11c (anti-CD11c-Alexa Fluor 647) for dendritic cells (BioLegend). Samples were washed twice and then examined using a Cytoflex flow cytometer (Beckman Coulter). Isolated cells were determined to be >90% pure.

### Differentiation of THP-1 cells.

THP-1 monocytes (6 × 10^6^) were differentiated to macrophage-like cells by treatment with 5 ng/ml phorbol myristate acetate (PMA) for 48 h, followed by a 24-h recovery period. Differentiation was confirmed visually by light microscopy as cells became adherent.

### Quantification of cytokines.

Cell-free culture supernatants were harvested at 8 h postinfection and frozen at −20°C. The levels of murine IL-6 and TNF-α and human TNF-α were measured using ELISA MAX kits (BioLegend) according to the manufacturer’s instructions. For murine IFN-β, the DuoSet ELISA kit (R&D systems) was used.

### Measurement of GAS adherence to macrophages.

Adherence to RAW264.7 cells was measured after 30 min of infection. Cells were gently washed three times with 500 μl PBS, followed by incubation with 100 μl trypsin for 5 min at 37°C, and then the addition of 400 μl of 0.1% saponin in sterile water for 1 to 2 min until cells appeared lysed under the microscope. Serial dilutions were plated on THY agar plates, and CFU were enumerated after overnight incubation at 37°C with 5% CO_2_.

### Measurement of GAS internalization by macrophages.

Viable internalized GAS was measured by gentamicin protection assay. Briefly, RAW264.7 or RAW-Blue cells were infected at an MOI of 10 for 30 min. Medium was aspirated and replaced with medium containing 100 μg/ml gentamicin. Cells were further incubated for 1 to 4 h. Wells were gently washed three times with 500 μl PBS, followed by incubation with 100 μl trypsin for 5 min at 37°C and then addition of 400 μl of 0.1% saponin in sterile water for 1 to 2 min until cells appeared lysed under the microscope. Serial dilutions were plated on THY agar plates, and CFU were enumerated after overnight incubation at 37°C with 5% CO_2_.

### Valspodar treatment of GAS.

GAS strains Δ*rgg2* and Δ*rgg3* were grown in CDM as described above. When cultures reached an OD of ∼0.1, they were supplemented with 10 μM valspodar (Sigma) and allowed to grow to an OD of ∼0.5. GAS cells were washed with PBS prior to inoculating macrophages.

### Statistical analysis.

Data were statistically analyzed using GraphPad Prism v.7.0b. Data were analyzed using ordinary one-way analysis of variance (ANOVA) followed by Tukey’s multiple-comparison test or two-way ANOVA followed by Sidak’s multiple-comparison test and deemed significant at *P* < 0.05 (*), *P* < 0.005 (**), *P* < 0.001 (***), and *P* < 0.0001 (****). ns is used to denote values that are not significantly different.
