# Supplementary figures and images for: A Streptococcus Quorum Sensing System Enables Suppression of Innate Immunity
Source: mBio. 2021 May 4;12(3):e03400-20. doi: 10.1128/mBio.03400-20 (PMC8262891; doi:10.1128/mBio.03400-20)

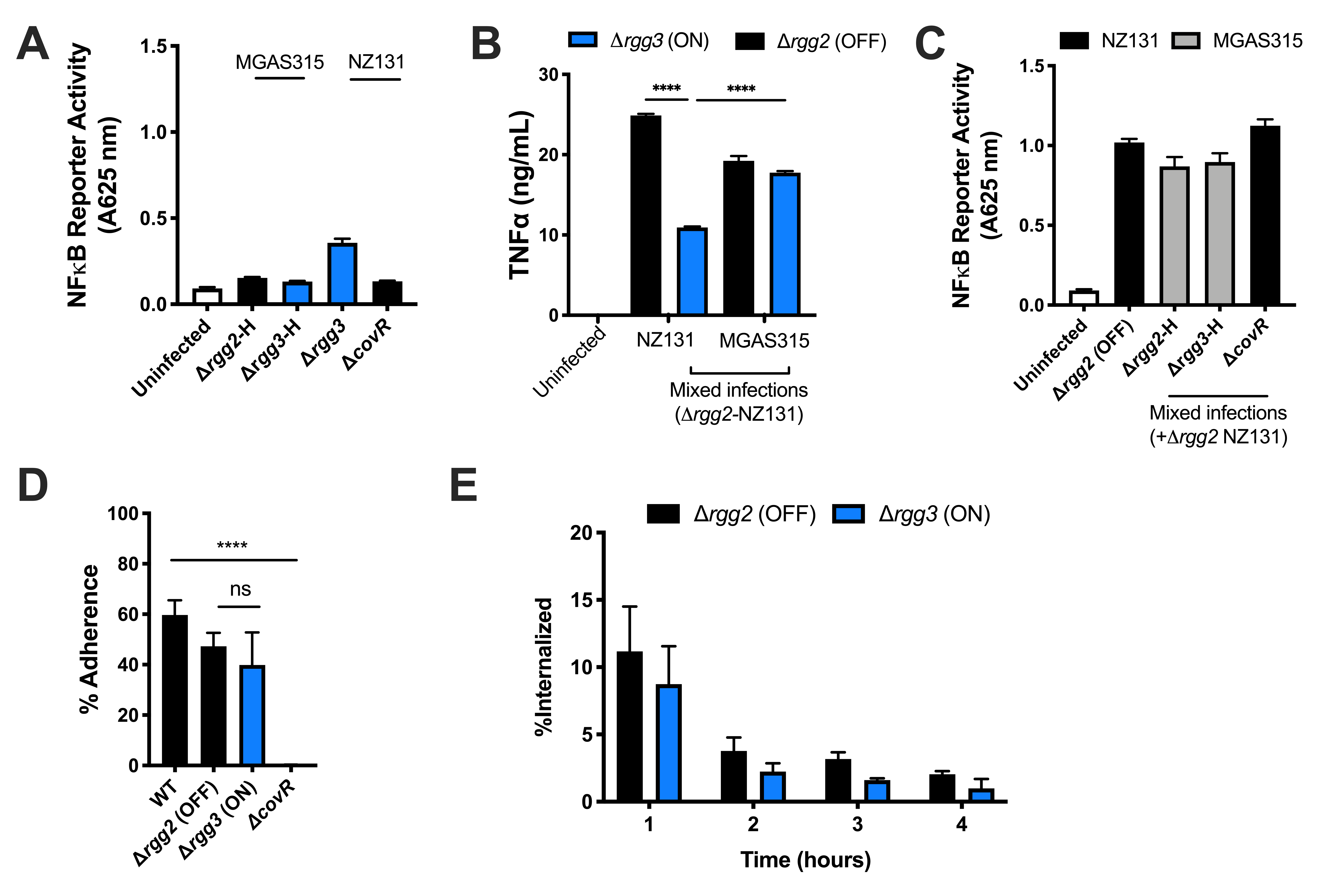

Supplement: FIG S1 [file mbio.03400-20-sf001.tif]
