# Supplementary material for: A Streptococcus Quorum Sensing System Enables Suppression of Innate Immunity
Source: mBio. 2021 May 4;12(3):e03400-20. doi: 10.1128/mBio.03400-20 (PMC8262891; doi:10.1128/mBio.03400-20)
Supplement: TABLE S1 [file mbio.03400-20-st001.docx]

**Supplemental Tables**

**Supplemental Table 1. Strains and plasmids used in this study**

| **Strain** | **Description** | **Reference** |
| --- | --- | --- |
| Δ*covR* | NZ131 Δ*covR*; unmarked | ^1^ |
| 5448 | Wild-type M1 isolate of the M1T1 lineage | ^2^ |
| HSC5 | Wild-type M14 isolate | ^3, 4^ |
| JCC131 | NZ131 Δ*rgg3*::*cat*; cm^R^ | ^5^ |
| JCC137 | NZ131 Δ*rgg2*; unmarked | ^5^ |
| JCC155 | NZ131 Δ*spy49_0450-0456*; unmarked | This study |
| JCC173 | NZ131 Δ*spyCEP*::*aphA3*; kan^R^ | This study |
| JCC303 | NZ131 Δ*spy49_0450-0460*; unmarked | This study |
| JCC304 | NZ131 Δ*spy49_0459*; unmarked | This study |
| JCC306 | NZ131 Δ*spy49_0460*; unmarked | This study |
| JCJ134 | NZ131 Δ*hasAB*::*cat*; cm^R^ | This study |
| JCJ173 | NZ131 Δ*stcA*; unmarked | ^6^ |
| JCC196 | MGAS315 Δ*rgg2*::*aphA3*; kan^R^ | ^7^ |
| JCC194 | MGAS315 Δ*rgg3*::*cat*; cm^R^ | ^7^ |
| MGAS315 | Wild-type M3 isolate | ^8, 9^ |
| NZ131 | Wild-type M49 isolate | ^10^ |
| RVW113 | NZ131 Δ*emm*::*aphA3*; kan^R^ | This study |
| RVW179 | NZ131 Δ*slo*_1-260_::*aphA3*; kan^R^ | This study |
|  |  |  |
| **Plasmid** | **Description** | **Reference** |
| p7INT | Shuttle-suicide vector that integrates at a streptococcal prophage tmRNA site; for complementation in single copy; erm^R^ | ^11, 12^ |
| pFED760 | Shuttle vector pGh9-ISS1 deleted for ISS1 element; used for constructing all deletion mutants; temperature-sensitive replication origin; erm^R^ | ^13^ |
| pJC202 | To construct unmarked deletion of *spy49_0450-0456*; in pFED760; erm^R^ | This study |
| pJC233 | Complementation of *spy49_0450-0457* in multi-copy; in pLZ12-Sp; sp^R^. *p0450-57* in text. | This study |
| pJC258 | To replace *spyCEP* with the *aphA3* kanamycin resistance marker; in pFED760; kan^R^ erm^R^ | ^1^ |
| pJC411 | To construct unmarked deletion of *spy49_0450-0460*; in pFED760; erm^R^ | This study |
| pJC412 | To construct unmarked deletion of *spy49_0459*; in pFED760; erm^R^ | This study |
| pJC414 | To construct unmarked deletion of *spy49_0460*; in pFED760; erm^R^ | This study |
| pJC420 | p7INT-based vector containing 74 bp of *shp3* promoter region; erm^R^ | This study |
| pJC469 | Complementation of *spy49_0459* in single copy; in pJC420; erm^R^. *p0459* in text. | This study |
| pJJ148 | To replace *hasAB* with the *cat* chloramphenicol resistance cassette; in pFED760; cm^R^ erm^R^ | This study |
| pLZ12-Sp | Shuttle vector for complementation in multi-copy; sp^R^ | ^14^ |
| pRVW48 | To replace the *emm49* region with *aphA3* kanamycin resistance marker; in pFED760; kan^R^ erm^R^ | This study |
| pRVW82 | To replace the N-terminal 260 amino acids of *slo* with the *aphA3* kanamycin resistance marker | This study |
|  |  |  |

cm = chloramphenicol; erm = erythromycin; kan = kanamycin; sp = spectinomycin

References

1. Wilkening, R. V., Chang, J. C. & Federle, M. J. PepO, a CovRS-controlled endopeptidase, disrupts Streptococcus pyogenes quorum sensing. *Mol. Microbiol.* **99**, 71-87 (2016).

2. Chatellier, S. *et al*. Genetic relatedness and superantigen expression in group A streptococcus serotype M1 isolates from patients with severe and nonsevere invasive diseases. *Infect Immun* **68**, 3523-3534 (2000).

3. Hanski, E., Horwitz, P. A. & Caparon, M. G. Expression of protein F, the fibronectin-binding protein of Streptococcus pyogenes JRS4, in heterologous streptococcal and enterococcal strains promotes their adherence to respiratory epithelial cells. *Infect Immun* **60**, 5119-5125 (1992).

4. Port, G. C., Paluscio, E. & Caparon, M. G. Complete Genome Sequence of emm Type 14 Streptococcus pyogenes Strain HSC5. *Genome Announc* **1** (2013).

5. Chang, J. C., LaSarre, B., Jimenez, J. C., Aggarwal, C. & Federle, M. J. Two group A streptococcal peptide pheromones act through opposing Rgg regulators to control biofilm development. *PLoS Pathog.* **7**, e1002190 (2011).

6. Gogos, A., Jimenez, J. C., Chang, J. C., Wilkening, R. V. & Federle, M. J. A quorum sensing-regulated protein binds cell-wall components and enhances lysozyme resistance in Streptococcus pyogenes. *J. Bacteriol.* (2018).

7. Gogos, A. & Federle, M. J. Colonization of the murine oropharynx by Streptococcus pyogenes is governed by the Rgg2/3 quorum sensing system. *Infect. Immun.* (2020).

8. Musser, J. M. *et al*. Streptococcus pyogenes causing toxic-shock-like syndrome and other invasive diseases: clonal diversity and pyrogenic exotoxin expression. *Proc Natl Acad Sci U S A* **88**, 2668-2672 (1991).

9. Beres, S. B. *et al*. Genome sequence of a serotype M3 strain of group A Streptococcus: phage-encoded toxins, the high-virulence phenotype, and clone emergence. *Proc. Natl. Acad. Sci. U. S. A.* **99**, 10078-10083 (2002).

10. McShan, W. M. *et al*. Genome sequence of a nephritogenic and highly transformable M49 strain of Streptococcus pyogenes. *J. Bacteriol.* **190**, 7773-7785 (2008).

11. Cho, K. H., Port, G. C. & Caparon, M. Genetics of Group A Streptococci. *Microbiol Spectr* **7** (2019).

12. McShan, W., McLaughlin, R., Nordstrand, A. & Ferretti, J. Vectors containing streptococcal bacteriophage integrases for site-specific gene insertion. *Methods Cell Sci* **20**, 51-57 (1998).

13. Mashburn-Warren, L., Morrison, D. A. & Federle, M. J. A novel double-tryptophan peptide pheromone controls competence in Streptococcus spp. via an Rgg regulator. *Mol. Microbiol.* **78**, 589-606 (2010).

14. Husmann, L. K., Scott, J. R., Lindahl, G. & Stenberg, L. Expression of the Arp protein, a member of the M protein family, is not sufficient to inhibit phagocytosis of Streptococcus pyogenes. *Infect Immun* **63**, 345-348 (1995).

stylefix
