# Supplementary material for: A Streptococcus Quorum Sensing System Enables Suppression of Innate Immunity
Source: mBio. 2021 May 4;12(3):e03400-20. doi: 10.1128/mBio.03400-20 (PMC8262891; doi:10.1128/mBio.03400-20)
Supplement: TABLE S2 [file mbio.03400-20-st002.docx]

**Supplemental Table 2. Primers used in this study**

| **Purpose** | **Primer** | **Sequence** | **Description** |
| --- | --- | --- | --- |
| pJC202 | JC195 | CATGgcggccgcAGAAGCTCAAGAGATGACC | S primer for upstream flanking region; NotI |
|  | JC133 | GAATTCTACACAGAGCTAACCATTACC | AS primer for upstream flanking region |
|  | JC193 | ctctgtgtagaattcAGATAGTGAAGGGTATTTTCG | S primer for downstream flanking region |
|  | JC194 | CATGgcggccgcCCAAACTGGCATCTAACC | AS primer for downstream flanking region; NotI |
| pJC233 | JC288 | GCATGggatccATATTTTCCCACTTTCCC | S primer for *spy49_0450*; BamHI |
|  | JC290 | GCATGggatccCCAAACTGGCATCTAACC | AS primer for *spy49_0457*; BamHI |
| pJC411 | JC575 | gaggatcccaccgcggtggcggccgcCAAGAACTTGTATGGCTTG | S primer for upstream flanking region |
|  | JC576 | tattctaaatactaacgcgtTACACAGAGCTAACCATTACC | AS primer for upstream flanking region |
|  | JC577 | acgcgtTAGTATTTAGAATATTATTTAAAAAGTATAATATTC | S primer for downstream flanking region |
|  | JC578 | ggatccactagttctagagcggccgcAGAGAGTCAATTAACAACTCTAAAC | AS primer for downstream flanking region |
| pJC412 | JC579 | gaggatcccaccgcggtggcggccgcAAACTATTAATGATAATTCTTTGAATC | S primer for upstream flanking region |
|  | JC580 | gtttttgttaacgcgtGTTAACCTCTATTCTAAAACTTTTTATAAATC | AS primer for upstream flanking region |
|  | JC581 | agaggttaacacgcgtTAACAAAAACATTTTTGCG | S primer for downstream flanking region |
|  | JC582 | ggatccactagttctagagcggccgcTATAAAATGGGGGAGCTAAC | AS primer for downstream flanking region |
| pJC414 | JC569 | ctctagaggatcccaccgcggtggcggccgcAACTAACCTAAAAGTTAATAAGCTTG | S primer for upstream flanking region |
|  | JC585 | tattctaaatactaacgcgtTTTAAAGACCTTTAAAATTTTCAG | AS primer for upstream flanking region |
|  | JC577 | acgcgtTAGTATTTAGAATATTATTTAAAAAGTATAATATTC | S primer for downstream flanking region |
|  | JC578 | ggatccactagttctagagcggccgcAGAGAGTCAATTAACAACTCTAAAC | AS primer for downstream flanking region |
|  | JJ13 | CATGagatctTGTCACCTAAATAGCTTGGC | S primer for inverse PCR on pJC420; BglII |
|  | JC592 | CATGgcggccgcCTTCCTTTCAGTTTTTATTTAT | AS primer for inverse PCR on pJC420; NotI |
| pJC469 | JC686 | CATGgcggccgcATGAAAATTACAGTTGTAGGC | S primer for *spy49_0459*; NotI |
|  | JC687 | CATGagatctTTCAGATATAATAACTTTTAACTG | AS primer for *spy49_0459*; BglII |
| pJJ148 | JC125 | CATGgtcgacGCGTTATTCAACAACATCG | S primer for upstream flanking region; SalI |
|  | JC122 | GATGAAGACATGGCTAAGGGACAATTACACCTCTTTC | AS primer for upstream flanking region |
|  | JC123 | ggtgtaattgtccctTAGCCATGTCTTCATCTATAAC | S primer for downstream flanking region |
|  | JC124 | CATGgcggccgcCTTTAAGAAGGGCTTGTAGG | AS primer for downstream flanking region; NotI |
| pRVW48 | RW143 | tggatcccccgggctgcaggaattcTGAAATGTTTTCTAACCACC | S primer for upstream flanking region |
|  | RW144 | tcattttagccatTGTTTACTCCTTATTTCTTAACTTTC | AS primer for upstream flanking region |
|  | RW147 | attgttttagGCCTTTAAAACTTGGTTTTTGTAACG | S primer for downstream flanking region |
|  | RW148 | taccgggccccccctcgaggtcgacAGCCTCAGGCATCGCACC | AS primer for downstream flanking region |
|  | RW145 | ggagtaaacaATGGCTAAAATGAGAATATCAC | S primer for *aphA3* |
|  | RW146 | aagttttaaaggcCTAAAACAATTCATCCAGTAAA | AS primer for *aphA3* |
| pRVW82 | RVW183 | tggatcccccgggctgcaggaattcGTCAACGATTGGTCGACAAG | S primer for upstream flanking region |
|  | RVW184 | tcattttagccatGTCCTTCATACCTTTTTATCATTCTAAAATG | AS primer for upstream flanking region |
|  | RVW274 | attgttttagTCACAGATTGAAGCAGCTC | S primer for downstream flanking region |
|  | RVW275 | taccgggccccccctcgaggtcgacCTACTTATAAGTAATCGAACCATATG | AS primer for downstream flanking region |
|  | RW185 | tatgaaggacATGGCTAAAATGAGAATATCAC | S primer for *aphA3* |
|  | RW273 | caatctgtgaCTAAAACAATTCATCCAGTAAAATATAATATTTTATTTTC | AS primer for *aphA3* |
